# 901. Integrated Community Health Screening for COVID-19 and HIV Promotes HIV Diagnoses and Linkage to Care

**DOI:** 10.1093/ofid/ofab466.1096

**Published:** 2021-12-04

**Authors:** Corey L Rosmarin-DeStefano, Eugene G Martin, Gratian Salaru, Barbara Tempalski, Diana Finkel, Eileen Scarinci

**Affiliations:** 1 North Jersey Community Research Inititiave (NJCRI), Denville, New Jersey; 2 Rutgers Robert W. Johnson Medical School, Somerset, New Jersey; 3 Rutgers University Diagnostic Laboratories Director, Laboratory RWJ University Hospital, Somerset, New Jersey; 4 NJCRI, Newark, New Jersey; 5 NJMS Rutgers University, Newark, NJ; 6 North Jersey Community Research Initiative (NJCRI), Newark, New Jersey

## Abstract

**Background:**

New Jersey experienced a 64% decrease in HIV screening during the COVID-19 pandemic, hampering the Federal “End the Epidemic Initiative”. From March 2020- May 2021, North Jersey Community Research Initiative, a community-based organization in Newark, NJ, noted a HIV seropositivity of 3.1% despite a decrease of 25% in testing. Qualitative interviews conducted virtually with community individuals and focus groups during that time period indicated that COVID-19 suggested clients were taking more risks due to feelings of isolation, depression and anxiety. NJCRI in collaboration with Robert Wood Johnson Medical School in Somerset, NJ and five other community-based partners in NJ wanted to assess if offering community combination COVID-19 screening and HIV screenings during the pandemic would increase community screening for HIV.

**Methods:**

CLIA Waived Screening for COVID-19 from two antigen assays, LumiraDx and BD Veritor was combined with a referenced laboratory based molecular screening from saliva Infinity Biologix under FDA emergency use authorization within CDC guidance with HIV Alere/Determine and INSTI in those individuals that identified as asymptomatic for COVID-19 but with high risk for HIV

**Results:**

NJCRI began the COVID-19 and HIV rapid screening to clients on January 4, 2021.Clients tested for COVID-19 (N=274), 3% tested positive for HIV and < 3% are self-reported HIV+ (94% of the sample tested negative for HIV). Overall, 92% of clients tested negative for COVID-19. Clients testing positive for COVID-19 (N=19), there was a 6% positivity rate utilizing COVID-19 Antigen by nasal swab. Those positive via COVID-19 Molecular (N=19) method, results indicate clients also tested positive 6% of the time using a saliva indicator. Approximately, 5% of the study sample are confirmed COVID-19 positives via both testing methods (separately 1% Antigen and < 2% Molecular). 19% of the sample (N=3) tested positive for both HIV and COVID-19.

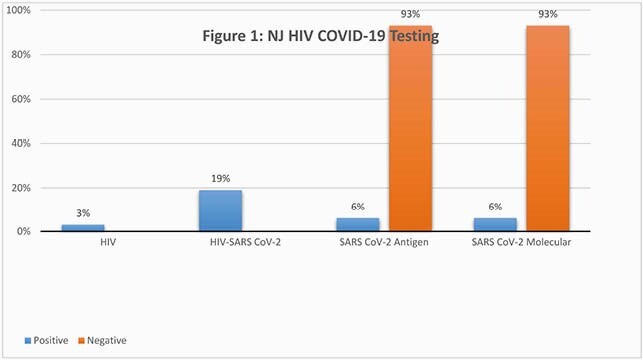

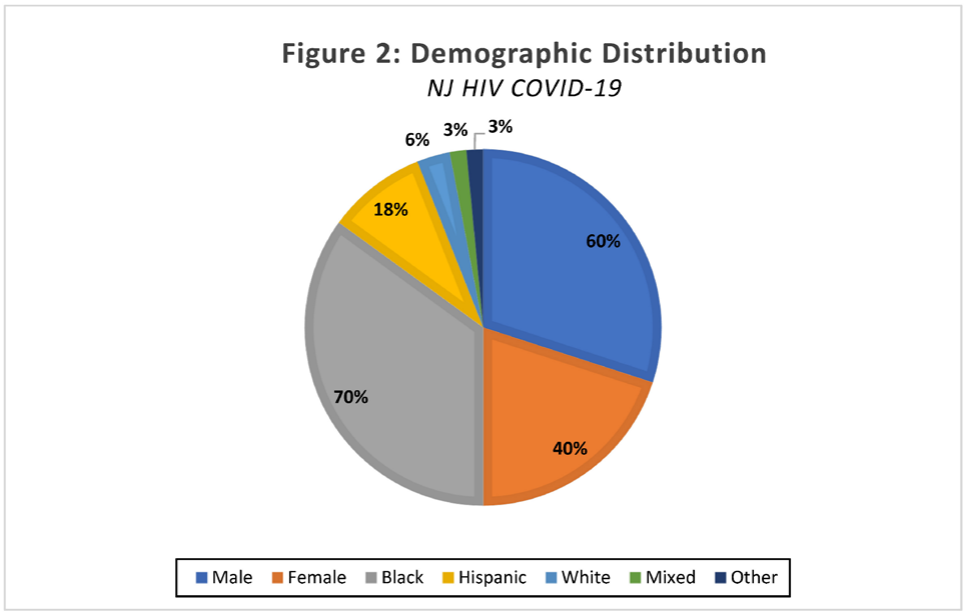

Figure 2. Demographics

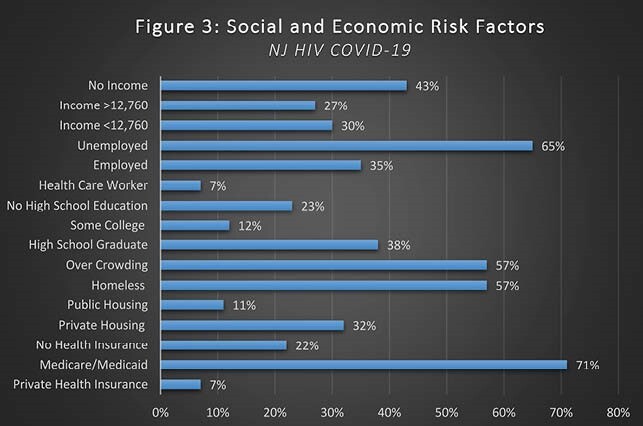

Figure 3. Social and Economic Risk Factors

**Conclusion:**

Newly diagnosed patients were treated the same day with antiretroviral therapy; linked to medical care, behavioral health and risk reduction services. Combining COVID-19 and HIV screening in a trusted community-based setting improved delivery of HIV care and linkage to care for newly diagnosed individuals in Newark, NJ.

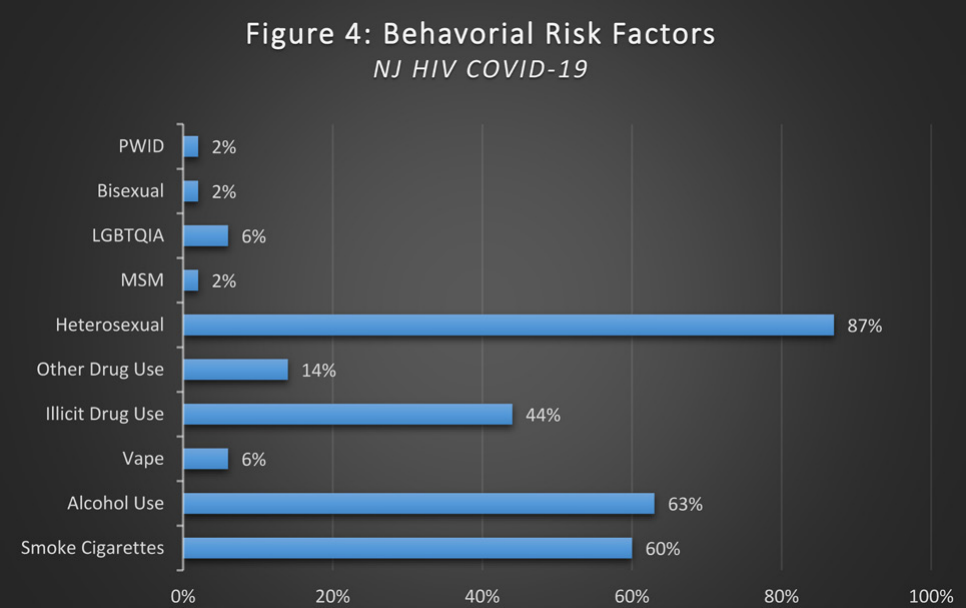

Figure 4. Behavioral Risk Factors

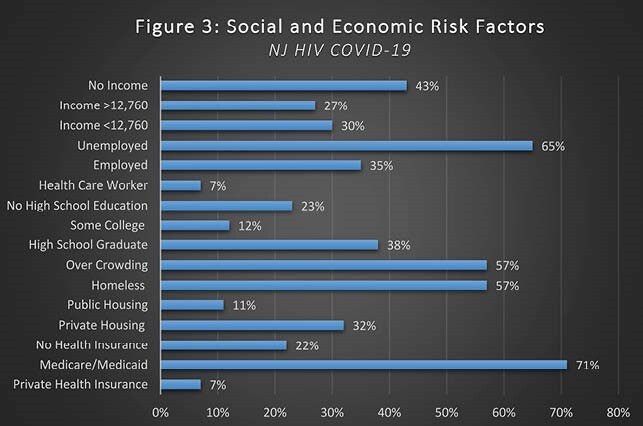

Figure 3. Social and Behavioral Risk Factors

**Disclosures:**

**All Authors**: No reported disclosures

